# An Auxin Transport-Based Model of Root Branching in *Arabidopsis thaliana*

**DOI:** 10.1371/journal.pone.0003673

**Published:** 2008-11-19

**Authors:** Mikaël Lucas, Yann Guédon, Christian Jay-Allemand, Christophe Godin, Laurent Laplaze

**Affiliations:** 1 IRD, UMR DIAPC (INRA/IRD/Montpellier SupAgro/UM2), Equipe Rhizogenèse, Montpellier, France; 2 INRIA, UMR DAP (CIRAD/INRIA/INRA/Montpellier SupAgro/UM2), Virtual Plants, Montpellier, France; 3 Université Montpellier II, UMR DIAPC (INRA/IRD/Montpellier SupAgro/UM2), Equipe Rhizogenèse, Montpellier, France; University of Nottingham, United Kingdom

## Abstract

Root architecture is a crucial part of plant adaptation to soil heterogeneity and is mainly controlled by root branching. The process of root system development can be divided into two successive steps: lateral root initiation and lateral root development/emergence which are controlled by different fluxes of the plant hormone auxin. While shoot architecture appears to be highly regular, following rules such as the phyllotactic spiral, root architecture appears more chaotic. We used stochastic modeling to extract hidden rules regulating root branching in *Arabidopsis thaliana*. These rules were used to build an integrative mechanistic model of root ramification based on auxin. This model was experimentally tested using plants with modified rhythm of lateral root initiation or mutants perturbed in auxin transport. Our analysis revealed that lateral root initiation and lateral root development/emergence are interacting with each other to create a global balance between the respective ratio of initiation and emergence. A mechanistic model based on auxin fluxes successfully predicted this property and the phenotype alteration of auxin transport mutants or plants with modified rythms of lateral root initiation. This suggests that root branching is controlled by mechanisms of lateral inhibition due to a competition between initiation and development/emergence for auxin.

## Introduction

Unlike animal development, plant development is essentially occurring post-embryonically. New organs are constantly derived from the activity of groups of undifferentiated cells called meristems that integrate both intrinsic developmental instructions and signals from the environment in which the plants is growing to give rise to an adapted architecture. Both the shoot and the root system depend on the functioning of meristems to develop branching structures. While the shoot architecture appears to be highly regular, following rules such as the phyllotactic spiral [1], the root architecture appears more chaotic and seems to be almost exclusively dependent on the environment. This might be the evolutionary consequence of the higher heterogeneity of the subterranean environment, compared to the above-ground conditions [2], [3] and suggests that shoot and root branching may be controlled by different mechanisms.

The plant hormone auxin is a key factor controlling lateral root formation from pericycle cells [4], [5]. Auxin controls both lateral root initiation [6], [7] and the development and emergence of lateral root primordia [8], [9], [10] but it is thought that while the initiation of lateral root primordia depends on auxin coming from the root tip (acropetal transport) [4], [9], their development and emergence depend on auxin flowing from the aerial part toward the root tip (basipetal transport) [8], [9], [11]. As lateral root primordia (LRP) arise from an inner root tissue (the pericycle), and are invisible until they eventually emerge [12], [13], the outward appearance of the root system does not reflect its internal structure. Recent studies tend to support the hypothesis that lateral root initiation is in fact more regular than initially thought [7], [14], [15].

In this study we used a combination of biological, stochastic and *in silico* modeling approaches to understand the mechanisms regulating root branching in the model plant *Arabidopsis thaliana*. We found that root branching shows macroscopic regularities at all times and at each structural level of the root system. We used stochastic modeling to extract rules followed by lateral root patterns. Among those, we observed the existence of feedback regulation between lateral root initiation and development/emergence. We designed a mechanistic model of root ramification integrating lateral inhibition due to competition for auxin. The predictions of the mechanistic model were confirmed by analyses of mutant plants altered in initiation or emergence. Lastly, we used gravistimulated plants to further study the balance between initiation and emergence and showed that gravistimulation enhances emergence. We were able to use the mechanistic model to reproduce the observed effect of gravistimulation on emergence.

## Results

### Arabidopsis root development exhibits order-independent, persistent macroscopic regularities

**Figure 1:**
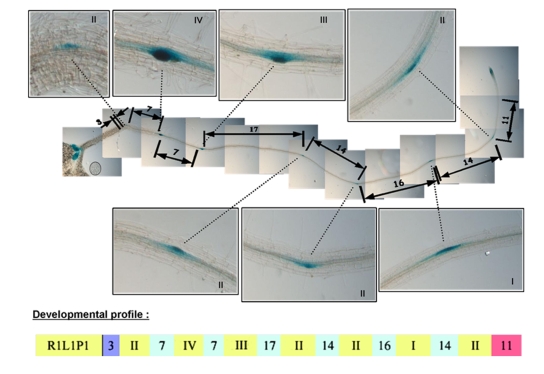
Encoding of root structure. Transgenic seedlings (n = 397) aged 3 to 12 day and expressing the *Pro_CYCB1_:GUS* marker were observed using a Leica DMRB microscope. The developmental stage of each primordium (indicated by Roman numerals) and the distance (measured in number of root hair cells) between them were scored along the primary root and emerged laterals. Each root was then assigned a unique identification code and developmental profile as illustrated here.

**Figure 2:**
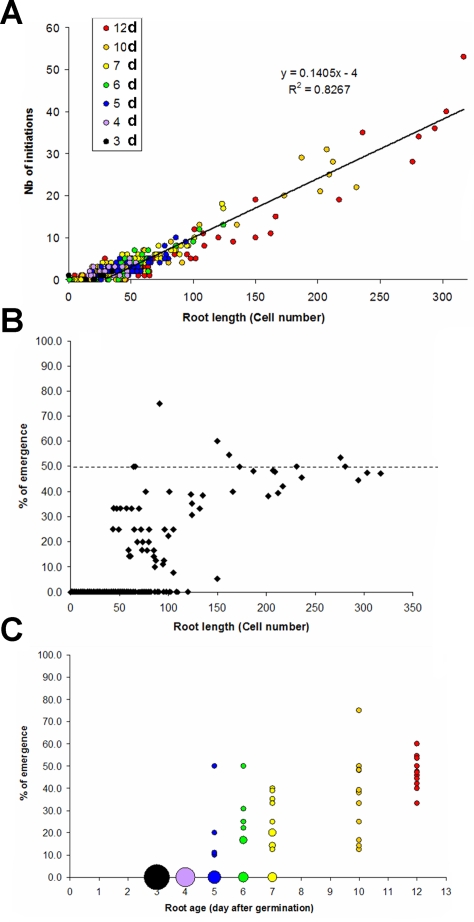
Macroscopic regularities of root development. Each data point corresponds to a single primary or secondary root (n = 397). The color of the point indicates the age of the root when observation took place (3 to 12 days after germination). (A) The global number of lateral root primordia initiation is proportional to the total root length (measured in number of root hair cells). (B) The global emergence rate of lateral roots stabilizes around 50%. (C) Stabilization of emergence rate occurs after the first week of growth. Data point size indicates the relative number of similar observed values.

In order to analyze the regulation of root architecture, we built and analyzed an extensive database of root developmental sequences of Arabidopsis seedlings. 400 *Col-0* seedlings aged from 3 to 12 days were observed and their developmental profiles were encoded as presented in Figure 1. It has been reported previously that mature Arabidopsis roots exhibit a stable mean number of lateral root primordia under controlled growth conditions [13], [14], [15]. The chronological analysis of root developmental profiles revealed a strong regularity in lateral root initiation rhythm expressed as a function of root length (expressed as a number of cells, Figure 2A). This initiation rhythm was stable for root aged from 3 to 12 days. Moreover, this regularity was order-independent, i.e. it was observed for primary roots as well as for secondary and tertiary roots of the 10 and 12-days old seedling (Figure 2A, orange and red data points for root length less than 150 cells long). While initiation appears to be highly regular from on the first stages of growth, emergence only appears stable after sufficient growth (Figure 2B). In our growth conditions, lateral root emergence seems to stabilize at around 50% after one week of growth (Figure 2C).

As such, these macroscopic observations show that initiation of new primordia follows a stable rhythm at each developmental stage and each branching order of the root system, whereas emergence might reach equilibrium after one week.

### Stochastic modeling of root development

While it was possible to observe strong regularities when studying large sets of roots at the macroscopic level, individual roots showed a high variability in developmental profiles at the microscopic level. We thus chose to use a stochastic model to extract structures from this large sample of root profiles. This made it possible to identify developmental patterns and regularities that were not directly apparent, due to the variability of the root architectures.

**Figure 3:**
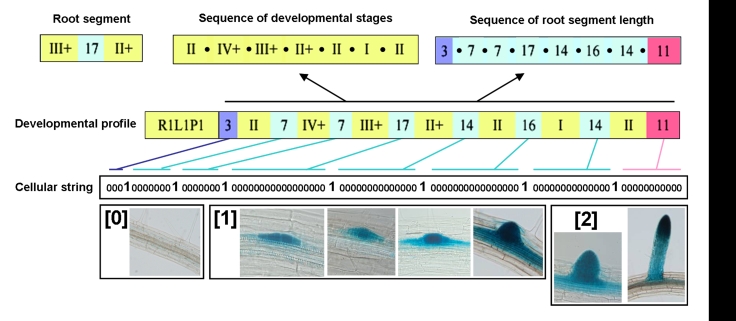
Encoding the root structure. We defined three kinds of sequences based on developmental profiles. The sequence of developmental stages considers only the developmental stages of the successive lateral organs. The sequence of root segment length (root segment being defined as the unit formed by two successive organs and the distance between them) considers only the distances between the successive lateral organs. The cellular string sequence were obtained by transcoding and expanding the developmental profile. The transcoding of the developmental stages is shown below the cellular string: observed un-differentiated cells were coded as 0, non-emerged primordia were coded as 1, and emerged lateral roots were coded as 2.

For the following database analysis, we defined a “root segment” as the developmental unit formed by two successive lateral organs (primordium or lateral root) and the distance between them, recorded as the observed number of epidermal (trichoblast) cells between the two lateral organs. From the developmental profiles composed by the succession of root segments, we extracted 3 types of developmental data for further analysis (Figure 3):

- sequences of developmental stages, considering only the developmental stages of the successive lateral organs,
- sequences of root segment lengths, considering only the distances between successive lateral organs,
- cell strings, resulting from the encoding of the full developmental profiles, with the following convention: 1 codes for non-emerged primordium, 2 codes for emerged lateral root and 0s indicate the segment length between two lateral organs.

**Figure 4:**
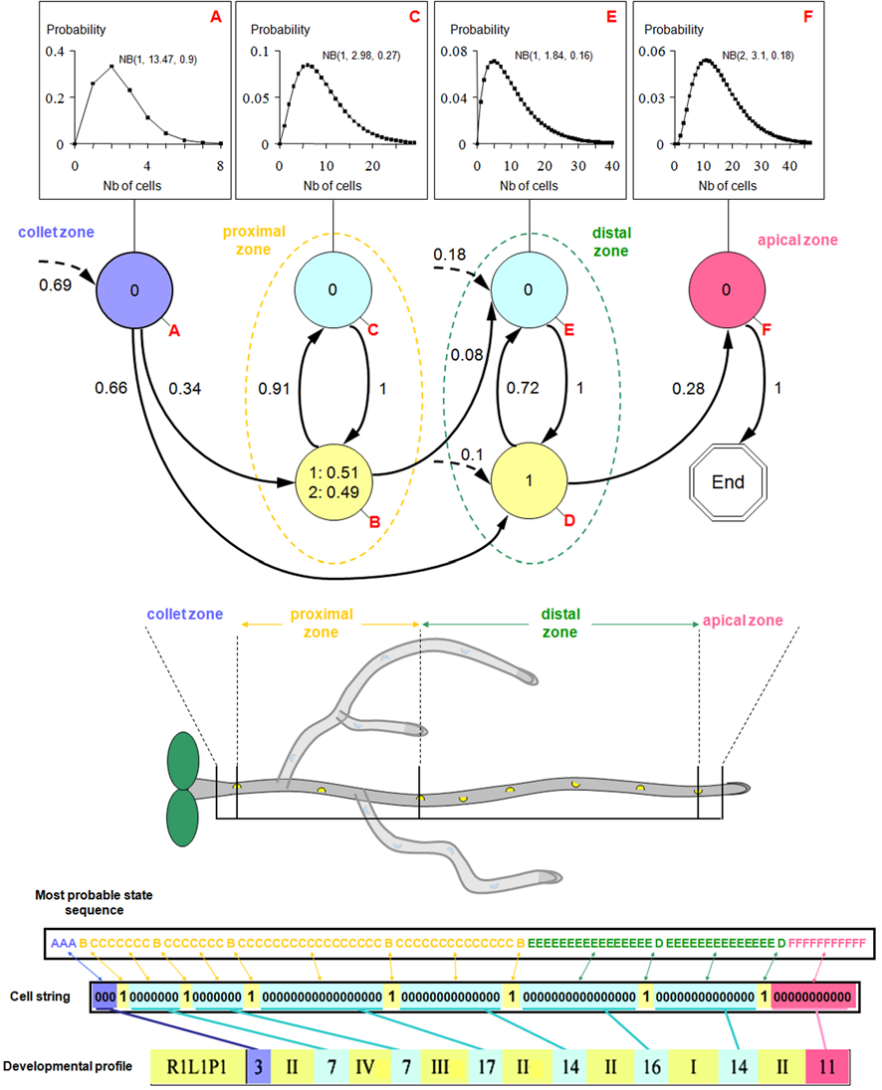
Stochastic model of root development. This model represents all observed developmental profiles (400 seedlings aged from 3 to 12 days). Each state is represented by a vertex which is labeled in red in its lower right corner (except the final end state). The possible transitions between states are represented by arcs with the attached probabilities noted nearby. Dotted arcs entering in states indicate initial states. The attached initial probabilities are noted nearby. Only arcs with attached initial or transition probabilities >0.03 are figured. The occupancy distributions of the semi-Markovian states A, C, E, and F are figured above the corresponding vertex. All these occupancy distributions are negative binomial distributions NB(*d*, *r*, *p*). The possible outputs in a state are noted in the corresponding vertex with the attached observation probabilities when <1. States B–C (respectively D–E) define the proximal (respectively distal) functional zones. The lower part of the panel present the most probable state sequence predicted for the given cell string.

The cell string transcription of the database was used as a basis to build a stochastic model based on Markov chains (see Materials and Methods for additional details). Such a model can be seen as an abstraction of the root developmental sequence (Figure 4) that efficiently summarizes all the observed root developmental profiles in a single unified model. The parameters were obtained by a classical likelihood maximization procedure [16], [17]. The estimated model is composed of 6 states (identified by the letters A–F). States A, C, E and F represent the segments (expressed as a sequence of cells) between lateral organs. The length of each segment is modeled by a distribution associated with each of these states (Figure 4, top row). States B and D represent the production of lateral organs. An additional terminal state was introduced to indicate the end of the sequence. In each state, outgoing arcs indicate the possible transitions from this state to the others. Each arc is associated with a probability that reflects the frequency of the corresponding transition in the cell string.

The model makes it possible to identify 4 main zones in roots from the collet to the root tip and to properly characterize these zones with, for instance, segment length distributions. The first zone (model state A) corresponds to the short developmental period following germination and preceding the first lateral root initiation (collet zone). The second macroscopic zone (proximal zone) is composed of state B (presence of a blocked primordium or of an emerged lateral root) and state C (root segment between two lateral organs) and corresponds biologically to the mature zone of the root. The third zone (“distal zone”) is composed of state D (presence of a primodium), and state E (root segment between two lateral organs) and corresponds to the zone of primordium development. The fourth zone (state F) corresponds to the developing root apex, where the next primordia will appear (apical zone). The estimated proportion of blocked primordia (51%) and emerged lateral roots (49%) in state B can be interpreted as an estimate of the emergence rate. The proximal zone (alternation of states B and C where it is only possible to enter this zone from state A with a probability of 0.34) is not always present while the distal zone (alternation of states D and E) is always present. This is a direct consequence of the fact that only roots that are older than 5 days after germination contain a mature zone.

**Figure 5:**
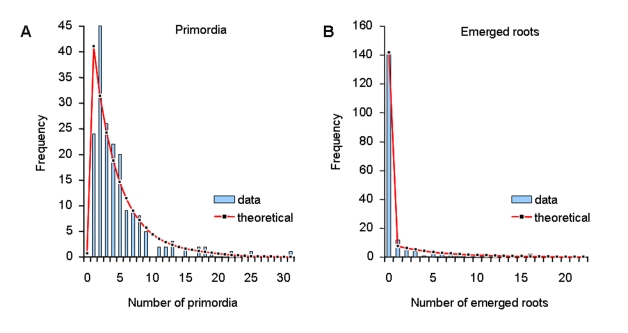
Primordia and lateral roots distributions in the stochastic model. The observed distributions (number of lateral organs of a given type per root) are extracted from the data while the theoretical distributions are computed from the estimated stochastic model.

Several theoretical distributions were computed from the estimated stochastic model and were compared to the corresponding empirical distributions. The predicted distributions of the number of lateral organs per root fitted well with the corresponding empirical distributions extracted from the observed data (*P*-value of 0.08 for primordia and 0.62 for emerged lateral roots with the Chi-square goodness of fit test) (Figure 5). This indicates that the stochastic model adequately captures the structure of the developmental sequences in the database.

### Stochastic model reveals interaction between primordium initiation and development

Along the complete root, lateral organs can be of three different types: developing LRP, blocked LRP and emerged lateral roots. In order to study the relationship between the distance and types of successive lateral organs, we had to restrict our analysis to regions where the fate of lateral organs was known (i.e. containing no developing LRP). Since lateral root initiation was shown not to occur between existing primordia [14], the analysis was made on proximal zones, where LRPs could be considered blocked in their development. The stochastic model was used to identify the proximal zone on each root by computing the most probable state sequence corresponding to the cell string. As a result, each cell string was optimally segmented into collet, proximal, distal and apical zones.

**Figure 6:**
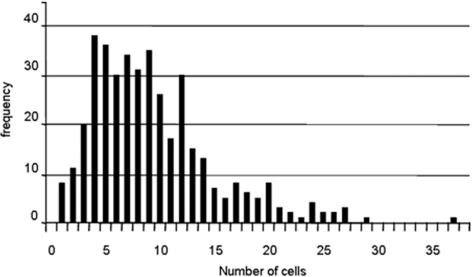
Empirical distribution of root segment length in the proximal zone (state 2 in the stochastic model).

We then analyzed the distribution of root segment lengths between lateral organs in the proximal zones (Figure 6). If the initiations were made at random and independently with respect to one another, one would expect geometric segment length distributions (the shorter the segment, the higher its frequency). However, in the observed distributions the most frequent segments have intermediate lengths and the shortest segments have low frequencies. This shows that the initiations are not independent of one another and could be explained by the existence of a lateral inhibition mechanism that prevents successive initiations at short distances.

**Figure 7:**
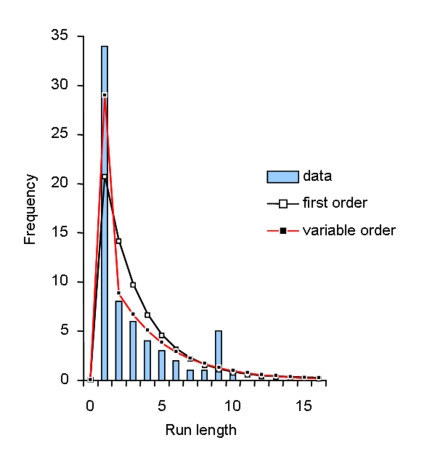
Distribution of the run length of emerged roots. The observed distribution (i.e. number of successive emerged roots) is extracted from the data while the theoretical distributions are computed from an estimated first-order or variable-order Markov chain.

We then considered whether particular rules were governing the emergence of lateral organs. We first wanted to know whether lateral root emergence events were interacting with each other and over which distance. For this, we built a variable-order Markov chain to model the succession of lateral organ types (blocked LRP or emerged LR denoted respectively by LRP, LR) in the proximal zones. Such a model makes it possible to automatically detect frequent patterns in the analyzed sequences while keeping a minimal number of parameters in the model. In our case, the observed sequences were best fitted by a variable-order Markov model of maximum order 2 (Figure 7). This model highlighted contrasted probabilities for successions of lateral organs ending by an emerged lateral root (Table 1):

i) at order 1 as LRP→LR (0.52) i.e. a blocked primordium was followed by an emerged lateral root in 52% of the cases.
ii) at order 2 as: LRP, LR→LR (0.55), i.e. an emerged root following a blocked primordium was followed by an emerged root in 55% of the cases; LR, LR→LR (0.76) i.e. two successive emerged roots were followed by an emerged root in 76% of the cases.

**Table 1:** Transition probabilities (with confidence intervals) of the estimated variable-order Markov chain.

	Next state		
Previous state(s)	primordium (LRP)	emerged root (LR)	count
Primordium (LRP)	0.48 (0.41, 0.55)	0.52 (0.45, 0.59)	197
primordium - emerged root (LRP - LR)	0.45 (0.34, 0.57)	0.55 (0.43, 0.62)	73
emerged root - emerged root (LR - LR)	0.24 (0.17, 0.32)	0.76 (0.68, 0.83)	127

While primordia appear to have a 50% chance of emerging if they are directly preceded by a non-emerged primodium, the probability of emergence rises to 0.76 if the two previous primordia both emerged. Hence, two modalities can be distinguished: the first where primordia and emerged roots tend to alternate (see the high frequency for value 1 in Figure 7) and the second with quite long runs of emerged roots (see the relatively long tail after the value 1 in Figure 7). Thus, the variable-order Markov chain analysis strongly supports the idea that successive lateral root emergence are not independent.

Then, to test whether the distance between successive lateral organs had an influence on the development of those organs, we studied the distribution of segment length before and after each organ depending on their type within the proximal zone. We found that the segments before and after a blocked primordium were significantly shorter than the segments before and after an emerged root according to the Student *t* test (*P*-value of 1.2 10^−6^; Table 2). As primordia positioning is sequential at the root apex, this correlation between organ development and position can be interpreted as the consequence of an inhibitory effect between primordia on lateral organ development. This effect tends to decrease as adjacent primordia are initiated farther away from each other.

**Table 2:** Empirical distributions of the segment length between two lateral organs.

*μ, σ (sample size)*	segment before	segment after
primordium	7.66, 5.6 (159)	8.74, 6.19 (191)
emerged root	10.4, 5.39 (243)	10.1, 5.91 (244)

### Design of a mechanistic model of lateral root initiation and development

**Figure 8:**
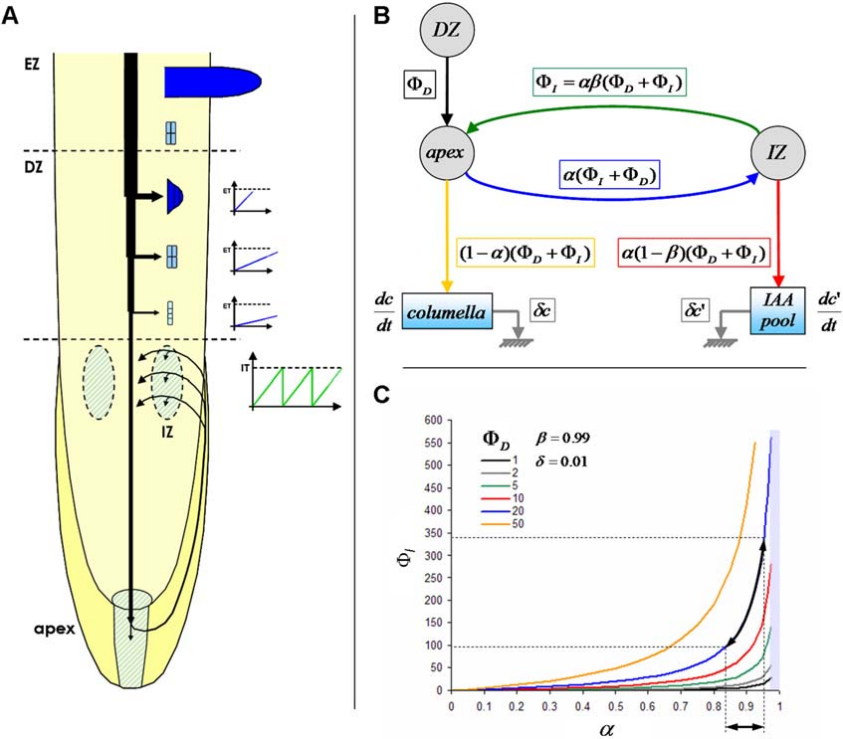
Mechanistic model of lateral root initiation and development. (A) Structure of the model. Auxin reflux takes place though the initiation zone (IZ). A fraction of the reflux accumulates until the initiation threshold (IT) is reached. A new primordium then appears and depletes auxin in the IZ. Primordia going through the development zone (DZ) drain a percentage of the central auxin flux. Primordia will emerge if their auxin content is higher than the emergence threshold (ET). Emerged laterals cease consuming auxin. Primordia which have not yet emerged when they leave the DZ for the emergence zone (EZ) will stop developing. Gravistimulation is considered to induce a drop of IT and to consume a fraction of the auxin in the IZ. IT and ET both vary dynamically according to Gaussian distributions. Auxin production augments after one week. (B) Mathematical representation of the reflux system. Fluxes coming from the development and initiation zone are denoted as Φ*_D_* and Φ*_I_*. The reflux efficiencies are denoted *α* and *β*, while *δ* expresses auxin degradation in the meristem and in the IZ. (C) Auxin fluxes passing through the IZ at equilibrium. As the reflux is considered to be imperfect, the flux going through the IZ will reach a stable point depending on the efficiencies of the refluxes *α* and *β* and on the central flux entering the IZ from the DZ (value in arbitrary units of production per minute). For high values of reflux efficiency, a small variation in reflux efficiency or entering fluxes will lead to a strong change of stable point (black arrows).

Our statistical analysis revealed an unexpected feedback between lateral root initiation and development. One possible explanation for this could be competition for auxin. To test this hypothesis, we designed a mechanistic model of root ramification based on auxin fluxes (Figure 8A). For this, we used a set of hypotheses which attempts to combine various knowledge sources coming from literature with our analysis in an integrated framework:

1 – Acropetal auxin fluxes come from the aerial parts, and increase after one week of growth [18]. The increase in auxin production after one week coincides with the acceleration of initiation rhythm we observed at the macroscopic level (Figure 2A – almost twice as many primordia appeared during the last 5 days than during the first 7).
2 – Developing primordia consume a fraction of the acropetal auxin flux according to an age-based hierarchy (older primordia have precedence over younger ones) [19].
3 – Primordia which have consumed enough auxin emerge (emergence threshold – ET) and stop consuming auxin [18], [19].
4 – After a given time (developmental window – DW), primordia which have not emerged are blocked and stop consuming auxin [14].
5 – The remaining auxin flux arrives at the root apex, where it takes part in a reflux system [7], [15], [20]. The mathematical properties of the reflux system (Figure 8B) lead to a stable point in flux intensity, which depends on the efficiency of the reflux at the apex and within the initiation zone, on the flux coming from the development zone, and on the auxin degradation rate (Figure 8C). For strong effective reflux values, the stable point flux in the initiation zone become increasingly responsive to small variation of parameters.
6 – Some of the auxin coming from the reflux flow through the lateral root cap and the epidermis and accumulates in the initiation zone (IZ) where initiation can take place. Initiation occurs when the level of auxin in this zone, considered as a pool of auxin, reaches a predefined initiation threshold (IT). The initiation of a new primordium empties the pool [7], [15], [19]. This assumption is based on biological data showing that lateral root initiation sites and young LRPs are auxin sinks and therefore consume auxin [21], [19].
7 – To account for the biological variability observed in the data, both the initiation and emergence thresholds (IT and ET) were submitted to random (Gaussian) fluctuations.
  In a previous study, we showed that initiation patterns can be significantly altered by controlled gravistimulation during root growth [15]. Such perturbations can be used to test the predictive power of the model. We therefore used the following hypotheses already tested by Lucas et al. [15] to take into account the effect of gravistimulation in the model:
8 – Gravistimulation induces the initiation of new primordia by reducing the initiation threshold (IT).
9 – Gravistimulation consumes a fraction of the auxin available for initiation (quick repetitions of gravistimulations inhibit initiation).
10 – Gravistimuli disappear over 4 hours, and as a consequence both the drop of IT and consumption of auxin in the IZ induced by a gravistimulus decrease regularly (linearly) over a 4 hour period.

**Figure 9:**
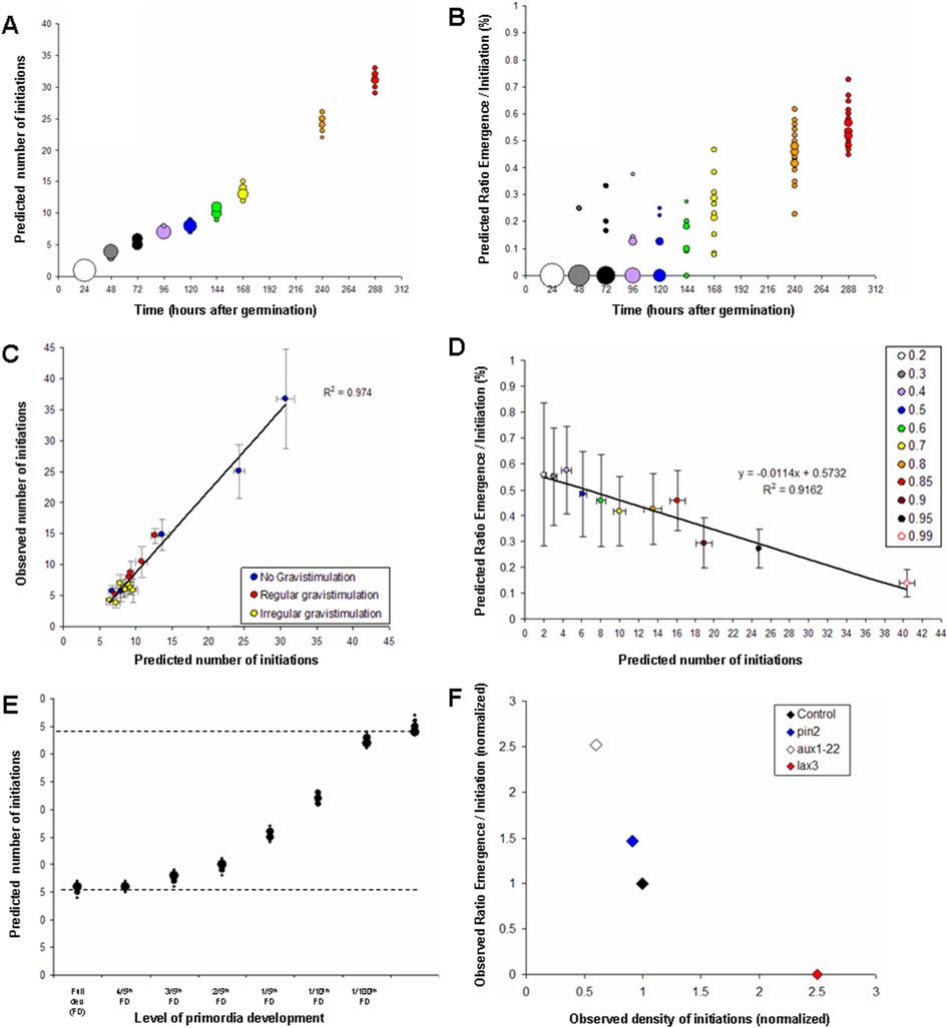
Mechanistic model calibration and predictions. (A & B) Initiation and emergence in the model were calibrated according to the observed mean initiation and emergence level (see figure 2). Runs of 20 simulations were done for each condition. Data point size indicates the relative number of simulations giving the same output. (C) Fit between model prediction and observation for various gravistimulation patterns (see Supplementary Figure S4 for additional details on the gravistimulation patterns). Runs of 20 simulations were done for each condition. (D) Predicted emergence rate and initiation level as a function of apical reflux. The color code indicates the reflux efficiency at the apex (reflux efficiency ranging from 20% to 99%). Runs of 20 simulations were done for each condition. (E) Predicted initiation level as a function of development level of primordia. Development of primordia in the model was either full or arrested at various level ranging from 4/5th of full development to no development at all. Runs of 20 simulations were done for each condition. Data point size indicates the relative number of simulations giving the same output. (F) Observed initiation and emergence densities in mutants and wild-type Col-0. Initiation and emergence densities were scored for the mutants *pin2* and *aux1*, and normalized in regard to the emergence density of wild-type Col-0 plants. Data for the *lax3* mutant were provided by Pr. Malcolm Bennett. Each data point corresponds to a set of more than 20 seedlings.

The corresponding computer algorithm is presented in Supplementary Figure S2. The parameters of the model were either directly extracted from observation (e.g. observed initiation rate, percentage of emergence, number of simultaneously developing primordia), or estimated through extensive parameter space exploration and comparison between model outputs and observation (see Supplementary Figure S3 for a detailed outlook of the reference used for parameters calibration). Due to the stochastic variation of IT and ET, each parameter set was tested by a run of simulations whose output were used for statistical comparison between observation and prediction. Figure 9 A & B shows the distribution of initiation and emergence obtained through calibration of the mechanistic model.

We were able to generate a set of parameters for which the model's output closely followed the observed number of lateral root initiation for normal root growth as well as for various gravistimulation patterns (Figure 9 A, B & C; Supplementary Figure S4).

### Mechanistic model predicts a balance between initiation and development

We used the mechanistic model to explore the potential interactions between primordium initiation and development. We first studied the effect of varying the reflux efficiency parameter from 20% to 99% and proceeded to the analysis of the resulting initiation and emergence levels. Since lateral root initiation is dependent on auxin coming from the apex via acropetal transport [7], the model predicted that a drop of reflux efficiency leads to a decrease of the initiation level and a concomitant increase of the emergence rate. For instance, if the reflux efficiency changes from 95% to 50%, the model predicts a 4-fold reduction of the initiation level and a 2-fold increase of the emergence/initiation ratio (Figure 9D). The positive effect on emergence could be attributed to a lowered competition for auxin between the less numerous initiated primordia. In order to validate this prediction, we used *Arabidopsis* mutants in which the auxin reflux mechanism is altered. Changes in the auxin reflux can be found in *pin2* and *aux1-22* mutants, in which the auxin reflux in the lateral root cap is reduced [20], [22], [23]. We compared the initiation and emergence densities of the *aux1-22* and *pin2* mutants to wild-type Col-0 seedlings. We found that mutants exhibited a rise in emergence level compared to the wild type, with a mean 2.5-fold increase in emergence for the most severe reflux reduction of *aux1-22* (Figure 9F).

We then studied the consequences of a premature arrest of primordium development on the initiation system in our model. Newly formed primordia were artificially arrested in their development (stopping auxin consumption from the central flux) when they reached a predefined development level. This analysis was done for decreasing development levels, ranging from full development to no development at all. The model predicted a roughly 2.5-fold increase of initiation level when primordium development was arrested as soon as new primordia appeared (Figure 9E). This was due to a lesser consumption of auxin by LRPs leading to a higher amount of auxin reaching the root tip and therefore transported acropetaly via the reflux system, thus enhancing the initiation rate. Premature arrest of primordium development can be found in another mutant of active auxin transporter, the *lax3* mutant [10]. This mutant exhibited a mean 2.5-fold increase in initiation levels compared to wild-type plants [10; Figure 9F].

### Balance between initiation and development is enhanced by gravistimulation

The consistency of our model predictions with different mutant phenotypes provides a first validation of the model's assumptions. In order to further test the concept of balance between initiation and development, we decided to artificially perturb the primordium initiation process and study the consequences on the development process.

**Figure 10:**
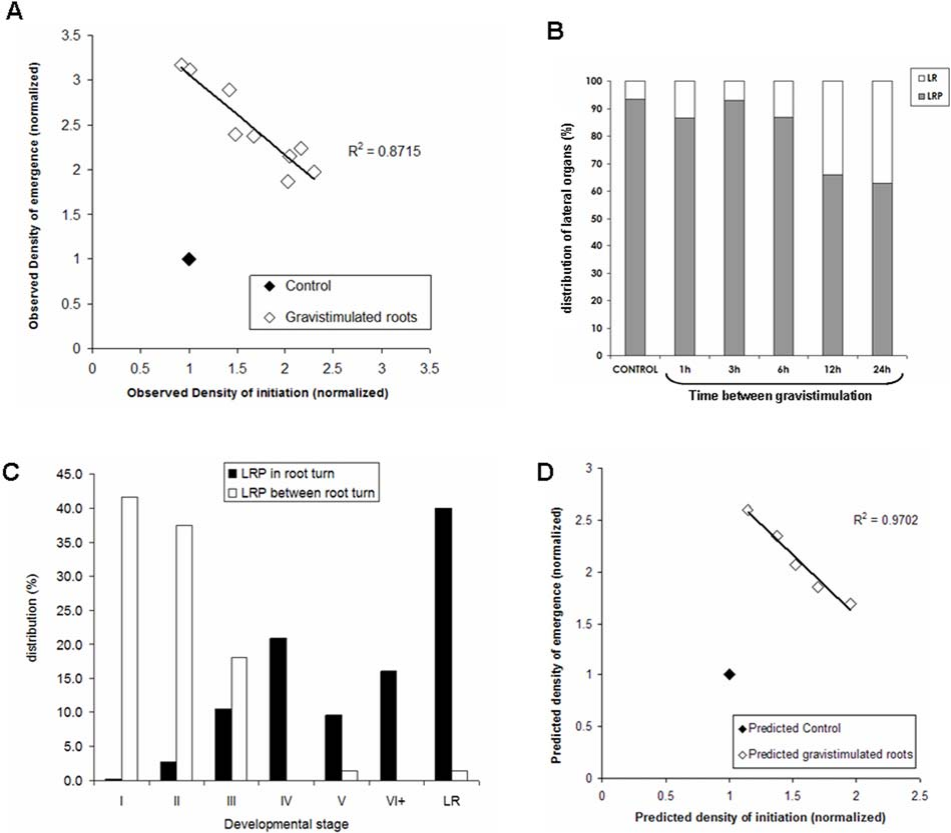
Gravistimulation enhanced balance between initiation and development. (A) Initiation density and emerged lateral root density were scored for plants gravistimulated according to the gravistimulation protocol presented in [15]. The results are given for primordia located in gravistimulated zones. Measurements were normalized in regard to the emergence density of non-gravistimulated plants. Each data point corresponds to a set of more than 20 seedlings. Non-gravistimulated Col-0 seedlings were used as a control group. (B) Emergence of lateral roots in gravistimulated roots. White bar: emerged lateral root percentage. Gray bar: non-emerged primordia percentage. Non gravistimulated Col-0 seedling were used as a control. (C) Distribution of primordia developmental stages for the 24 h time between gravistimulation treatment. White bar: primordia appearing and developing between gravistimulation (n = 72). Black bar: primordia appearing and developing in root turns (n = 373). (D) Initiation and emergence densities predicted by the mechanistic model with the added hypothesis of a drop of ET under gravistimulation.

We used a system of gravistimuli-induced initiation previously described [15]. This system allows for a wide range of controlled alteration of initiation level. We applied regular gravistimulation patterns with a time between rotations ranging from 1 to 24 hours. As a result, the initiation and emergence densities observed in the gravistimulated zones were negatively correlated (Figure 10A). This is consistent with the existence of a balance between initiation and emergence that was predicted by the model and observed in the mutant analysis. In addition, gravistimulated roots presented a homogeneous rise in emergence rate (Figure 10A). To elaborate on this, we compared the development of lateral root primordia for different gravistimulation rhythms (Figure 10B). We observed that, independently from the gravistimulation pattern applied, seedlings which were stimulated with low level of gravistimulation (12 h and 24 h between gravistimulation) presented a four-fold increase in percentage of emerging lateral organs. As the seedlings gravistimulated every 24 h presented primordium initiation in-between root gravistimulations, we were able to directly compare the development of primordia occurring during or outside of gravistimulus (Figure 10C). The observed distribution of developmental stages showed a clear distinction between those two primordia populations. Primordia initiated by gravistimulations at root bends developed faster (and/or more strongly) than primordia occurring outside bends.

We integrated this new effect due to gravistimulation in the mechanistic model by assuming that gravistimulation facilitates the emergence of lateral roots. This was translated in the model as a drop of ET for primordia formed during gravistimulation. This single hypothesis was sufficient to reproduce the negative linear correlation between the initiation and emergence densities due to gravistimulation, with comparable variation amplitudes (Figure 10D).

## Discussion

In this study, we used a combination of stochastic analyses, *in silico* modeling and biological observations to study the mechanisms regulating root branching in the model plant *Arabidopsis thaliana*. A large number of roots (400) from plants aged from 3 to 12 days were analyzed and encoded to generate a database of developmental profiles. The study of this database indicated that while lateral root initiation and development shows strong macroscopic regularities, there was a large variability between individual roots. We therefore used a stochastic model to extract a succinct set of probabilistic rules that capture this diversity. This stochastic model demonstrated that root branching is strongly structured and follow some developmental rules. Moreover it suggests the existence of a feedback regulation between lateral root primordium initiation and development/emergence. This was an unexpected finding as those two phenomena occur in distinct parts of the root. Moreover, while auxin is the main regulator of both lateral root initiation [6] and development/emergence [9], [10], these two processes were shown to depend on different auxin fluxes. [4], [7], [10], [24]. Lateral root initiation is regulated by auxin coming form the root tip (acropetal transport) [6], [7], [15] while lateral root development/emergence depends on auxin coming from the shoot (basipetal transport).

We generated a novel mechanistic model of root branching based on auxin fluxes that was able to reproduce the observed competition between primordium initiation and development. This model was also found to accurately predict the phenotype of mutants perturbed in root acropetal auxin transport (*pin2*, *aux1-22*) and lateral root emergence (*lax3*). The feedback between lateral root initiation and development/emergence was consistent with our analysis of plants with modified rates of initiation using gravistimulations [15], with the added observation that emergence was enhanced by gravistimulation. We showed that a simple extension of the mechanistic model was sufficient to predict the effect of gravistimulation on lateral root initiation and development/emergence. Moreover, the global qualitative behaviour of the model (balance between initiation and emergence) was preserved over a large range of parameters values, supporting the idea that the behaviour of the system itself derives from its governing rules and concepts. However, our mechanistic model does not explain the occurrence of either patches of emerged lateral roots or the alternation of LRPs and emerged lateral roots that we detected with our stochastic model.

Additional studies will need to investigate the enhancement of lateral root development by gravistimulation. The higher emergence rate observed in root turns may be linked with the mechanical constraints existing within the outer tissue layer. It has been shown that emergence is linked with a remodeling of the cell walls in the endoderm, cortex and epidermis, allowing the emerging primordia to push through the outer tissue layer without tearing up the surrounding cells [10]. Differential cell elongation occurring during root bending would theoretically induce longitudinal strains on the tissue, facilitating dissociation of cell walls in the same zone where the primordium appears and will potentially emerge. Potential experiments to investigate this hypothesis include physical measurements of the strains existing within root turns and study of the emergence level in root turns for emergence mutants such as *lax3*.

Taken together, our findings indicate that even if lateral root initiation and development/emergence are dependent on different auxin fluxes in the root, they use the same limited pool of auxin thus creating feedback mechanisms. These mechanisms are akin to inhibitory fields as defined by Hofmeister [25]. Inhibitory fields were historically proposed as a theoretical explanation for the phyllotaxis arising from the shoot apical meristem [26]. It has been shown in the last few years that the inhibitory fields regulating phyllotaxis, and subsequently shoot branching, were related to auxin and auxin transport [27], [28], [29]. Root branching however was known to be regulated by auxin since the discovery of the hormone itself, but no regular mechanism was ever proposed to explain how auxin directs root branching. Our findings suggest that regulation of root and shoot branching by auxin share common theoretical bases, pointing to potentially unified molecular mechanisms of plant development.

Mechanistic modeling proved to be a powerful tool to integrate and test biological concepts that would be too complex to comprehend otherwise. As our knowledge of auxin flux regulation grows, the opportunities to use mechanistic modeling to study auxin transport occurring at the cellular level during lateral root initiation multiply. We are currently developing an *in silico* cellular model of auxin fluxes to understand how the redistribution of auxin in the root apex may control the fine positioning of lateral root initiation.

## Materials and Methods

### Plant material and growth conditions

Wild type (Col-0), *pin2* and *aux1-22* mutants (Col-0 background) seeds were obtained from the NASC. *Pro_CYCB1_:GUS* (Col-0 background) seeds were provided by Dr P. Doerner (University of Edinburgh, UK). Plants were grown on vertical plates as previously described [30]. When applied, gravistimulations consisted of 90° successive rotations of the plates. For additional details on the periodical gravistimulation, see [15].

### Microscopy

Seedlings were collected and incubated in a solution containing 50 mM sodium phosphate buffer, pH 7.0, 0.5 mM K_3_Fe(CN)_6_ and K_4_Fe(CN)_6_, 0.05% (v/v) Triton X-100, 0.05% (v/v) DMF, 0.02% (v/v) EDTA, and 1 mM 5-bromo-4-chloro-3-indolyl-β-glucuronic acid and incubated at 37°C for several hours. Seedlings were then cleared in 70% (v/v) ethanol for 24 h, before being immersed for 2 h in 10% (v/v) glycerol 50% (v/v) ethanol; 2 h in 30% (v/v) glycerol 30% (v/v) ethanol; 2 h in 50% (v/v) glycerol. Seedlings were mounted in 50% (v/v) glycerol and visualized using a DMRB microscope (Leica). Pictures of the plants were obtained using a MZFLIII (Leica) dissecting microscope equipped with a digital camera.

### Observation

Development stages of successive lateral organs (as defined by [12]) and the distances between them (i.e. number of epidermal hair cells) were scored using the previously mentioned optical microscope. Special care was taken to follow continuous epidermal cell file in scoring the distance between successive lateral organs. When root spiraling became too important to allow the observation of a single continuous epidermal cell file, observation was resumed on a visible cell file in phase with the previous one. Cell counting always started at the collet, and cell counting ended when protoxylem ladder-structure was no longer visible.

Basic statistical analyses were made using the Excel statistical package.

### Stochastic model

We chose to model the cell string structure by a specific hidden Markovian model. Our model incorporates four semi-Markovian states with attached occupancy distributions to model the four types of segments of epidermal cells between lateral organs and two Markovian states to model the occurrence of either primordia or emerged roots in the proximal and distal zones. In this study, we assumed that the end of an observed sequence systematically coincides with the transition from the last segment state to an extra absorbing “end” state. The estimated hidden hybrid Markov/semi-Markov chain is shown in Figure 4. The model is “hidden” since epidermal cells (output 0) can be observed in the four semi-Markovian states while both primordia (output 1) and emerged roots (output 2) can be observed in Markovian state B (hence, the observed cell differentiation stage does not enable to determine the state in the model). The resulting hidden hybrid Markov/semi-Markov chain is thus defined by four subsets of parameters:

- Initial probabilities to model which is the first state occurring in the primary root,
- Transition probabilities to model the succession of states along the primary root,
- Occupancy distributions attached to semi-Markovian states to represent the segment length in number of epidermal cells,
- Observation distributions to model the composition properties within a state. All the observation distributions are degenerate i.e. a single output can be observed in a state except the observation distribution for the Markovian state B with a mixture of primordia and emerged roots.

The maximum likelihood estimation of the parameters of a hidden hybrid Markov/semi-Markov chain requires an iterative optimization technique, which is an application of the expectation-maximization (EM) algorithm [16], [17]. The hidden hybrid Markov/semi-Markov chain was estimated on the basis of 185 sequences of cumulated length 14,065. These sequences correspond to the roots showing at least one primordium. The 20 parameters consist of 3 independent initial probabilities, 4 independent transition probabilities, 12 parameters for the occupancy distributions attached to the four semi-Markovian states (all these occupancy distributions are negative binomial distributions NB(*d*, *r*, *p*) where *d* is an integer-valued shift parameter *d*≥1, *r* a real-valued shape parameter (*r*>0) and *p* a probability (0<*p*≤1)), and 1 independent observation probability (Markovian state B). Once the hidden hybrid Markov/semi-Markov chain had been estimated, the most probable state sequence was computed with the Viterbi algorithm [17] for each observed sequence. On the basis of this global stochastic model of cell string structure, various sub-samples and data characteristics were extracted and analyzed.

All the statistical analyses were made using the VPlant statistical package (successor of AMAPmod [31]) integrated in the OpenAlea platform [32], available at http://openalea.gforge.inria.fr/wiki/doku.php?id=openalea.

### Variable order Markov chain modeling

The succession of blocked primordia and emerged roots was analyzed within the proximal zone using variable-order Markov chains. In variable-order Markov chains, the order (or memory length) is variable and depends on the context within the sequence instead of being fixed. We applied the algorithm proposed by [33] for estimating variable-order Markov chains. This algorithm both selects the optimal set of memories and estimates the transition probabilities attached to each memory (for instance the transition probabilities attached to the second-order memory “primordium, emerged root”). Variable-order Markov chains of maximum order 3 were compared on the basis of 43 proximal zones of long root sequences (cumulated length 445). The variable-order Markov chain with memories “primordium” (order 1), “primordium, emerged root” and “emerged root, emerged root” (order 2) was selected; see Table 2 for the estimated transition probabilities with associated confidence intervals. Compared with a fixed first-order Markov chain, the fit of the run length of emerged roots was greatly improved (Figure 7).

### Mechanistic modeling

The root branching process was formalized as a mechanistic model (Figure 8A). Let Φ*_D_* and Φ*_I_* be respectively the flux entering the apex from the development zone and the reflux returning to the apex from the initiation zone (Figure 8B). Let *α* (respectively *β*) be the fraction of the auxin flux leaving the apex (respectively the initiation zone) toward the initiation zone (respectively the apex), with *α*, *β*∈]0;1[. Hence the accumulation of auxin within the meristem (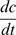) and within the initiation zone (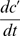) can be written as:
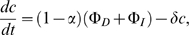
(1)

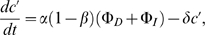
(2)where *δ* is the degradation rate of auxin.

The conservation of fluxes at the IZ node leads to:
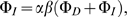
(3)Isolating Φ*_I_* in the left-hand term yields:
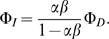
(4)From (1), (2) and (4) we can express the accumulation of auxin within the meristem and initiation zone as:
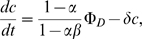
(5)

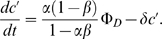
(6)We can thus express the auxin concentration within the meristem and initiation zone at equilibrium:
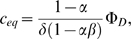
(7)

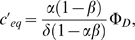
(8)and integrate (5) and (6) as:
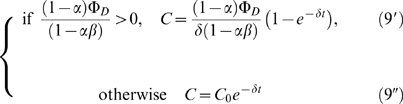


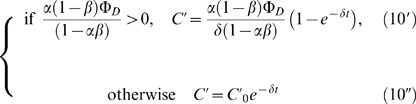
where *C*_0_ and 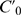 are the initial auxin concentrations within the meristem and initiation zone. As *α*, *β*∈]0;1[, equations (9′) and (10′) are true as long as Φ*_D_*>0.

Supplementary Figure S1 compares the values of C and C′ for different values of *δ* and Φ*_D_*.

The equation (4) shows that Φ*_I_* is particularly sensitive to values of *αβ* that are close to 1. For such values, the system shows large variations in Φ*_I_* even for small changes in either *αβ* or Ф*_D_* (Figure 8C).

A discrete version of this model written in the Python programming language is given in Supplementary Figure S2.

The different model parameters were either estimated directly from observed data (e.g. mean time between successive initiations, mean percentage of emergence, simultaneous number of developing primordia) or inferred from observed data through automated parameters' space exploration (see Supplementary Figure S3 for additional details). Over 1000 parameter combinations were tested. Due to the stochastic distribution of IT and ET, each parameter combination was tested for run of 20 simulations, and outputs were used for statistical evaluation of the parameter set. The parameter combination corresponding to the best fit of lateral root initiation and emergence densities to the observed values was selected for subsequent model prediction. The Python stand-alone module is available from the authors.

## Supporting Information

Figure S1 Auxin accumulation in the meristem and initiation zone (Top row) Auxin accumulation during time within the meristem (C) and initiation zone (C′) as a function of auxin fluxes coming from the differentiation zone, computed according to the reflux system presented in Figure 8B. α = 0.9 ; β = 0.9 ; δ = 0.01. Auxin level is expressed in arbitrary units. (Bottom row) Auxin accumulation during time within the meristem (C) and initiation zone (C′) as a function of auxin degradation, computed according to the reflux system presented in Figure 8B. α = 0.9 ; β = 0.9 ; ΦD = 10. Auxin level is expressed in arbitrary units.
(0.29 MB TIF)

Figure S2 The RootFeedback algorithm corresponding to the mechanistic model The pseudo-code is expressing the mechanisms described in Fig. 8A in discrete time.
(0.14 MB TIF)

Figure S3 Mechanistic model parameters choice. Parameters α, δ and IT were chosen a priori for simplicity sake. The developmental_window and return_time parameters were derived directly from the fact that emergence occurred as early as 5 days and that gravistimulated roots need 4 hours to bend in the new direction of the gravity vector. All others parameters were chosen arbitrarily and then refined a posteriori through iterative simulations to fit with observed values of initiation and emergence levels for 3 to 12 days old seedlings.
(0.17 MB TIF)

Figure S4 Gravistimulation patterns used for the calibration and evaluation of the model Seedlings were grown in vertical plates and gravistimulated by a 90° rotation (black dot) of the growth plates. Treatments labeled 1 to 24 were applied for 3.5 days using either crenel-shape-generating or stair-shape-generating protocols (see [15] for additional details). The results obtained following those treatments were use to calibrate the model presented in figure 8. Treatment labeled A to I were applied for 48 hours after germination using stair-shape-generating protocols. The results obtained following those treatments were directly compared to model output using the parameters previously defined.
(0.27 MB TIF)
