# Supplementary figures and images for: An Auxin Transport-Based Model of Root Branching in Arabidopsis thaliana
Source: PLoS One. 2008 Nov 19;3(11):e3673. doi: 10.1371/journal.pone.0003673 (PMC2577305; doi:10.1371/journal.pone.0003673)

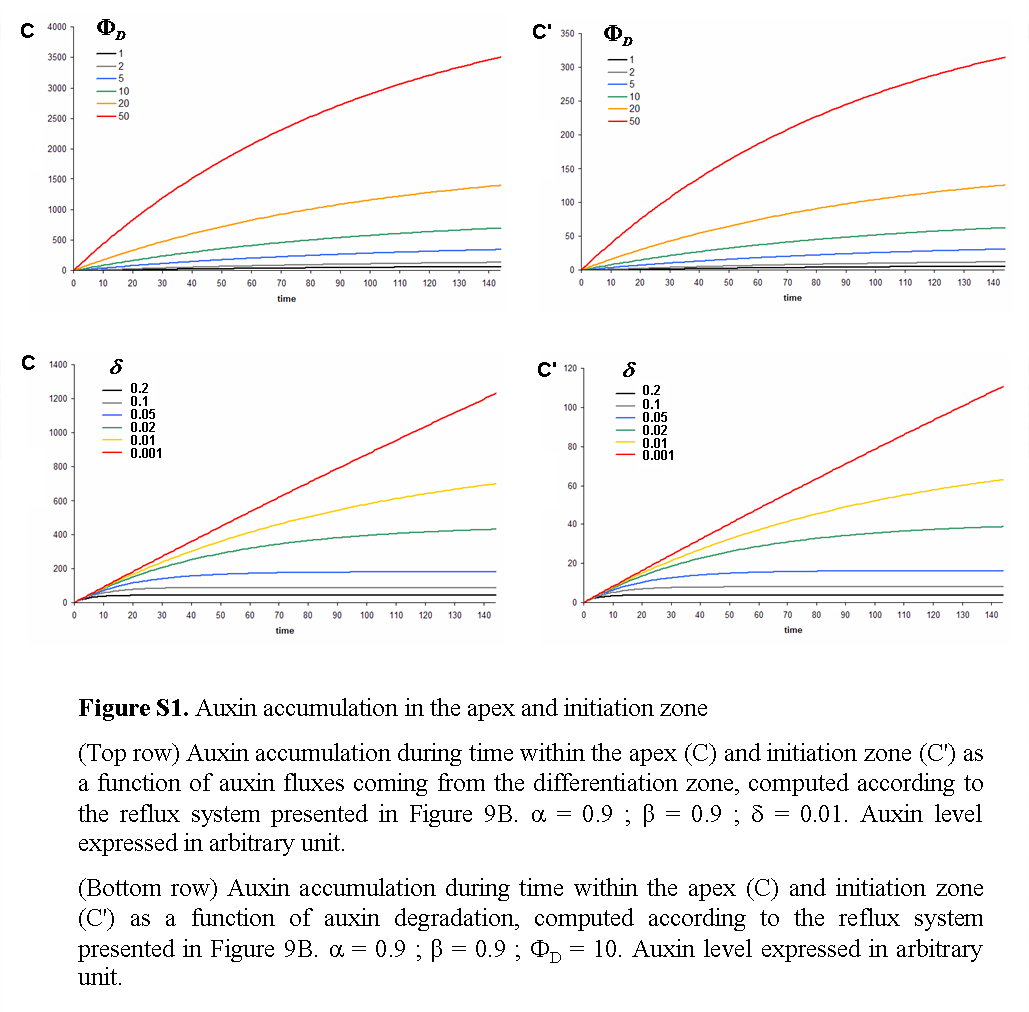

Supplement: Figure S1 — Auxin accumulation in the meristem and initiation zone (Top row) Auxin accumulation during time within the meristem (C) and initiation zone (C′) as a function of auxin fluxes coming from the differentiation zone, computed according to the reflux system presented in Figure 8B. α = 0.9 ; β = 0.9 ; δ = 0.01. Auxin level is expressed in arbitrary units. (Bottom row) Auxin accumulation during time within the meristem (C) and initiation zone (C′) as a function of auxin degradation, computed according to the reflux system presented in Figure 8B. α = 0.9 ; β = 0.9 ; ΦD = 10. Auxin level is expressed in arbitrary units. (0.29 MB TIF) [file pone.0003673.s001.tif]

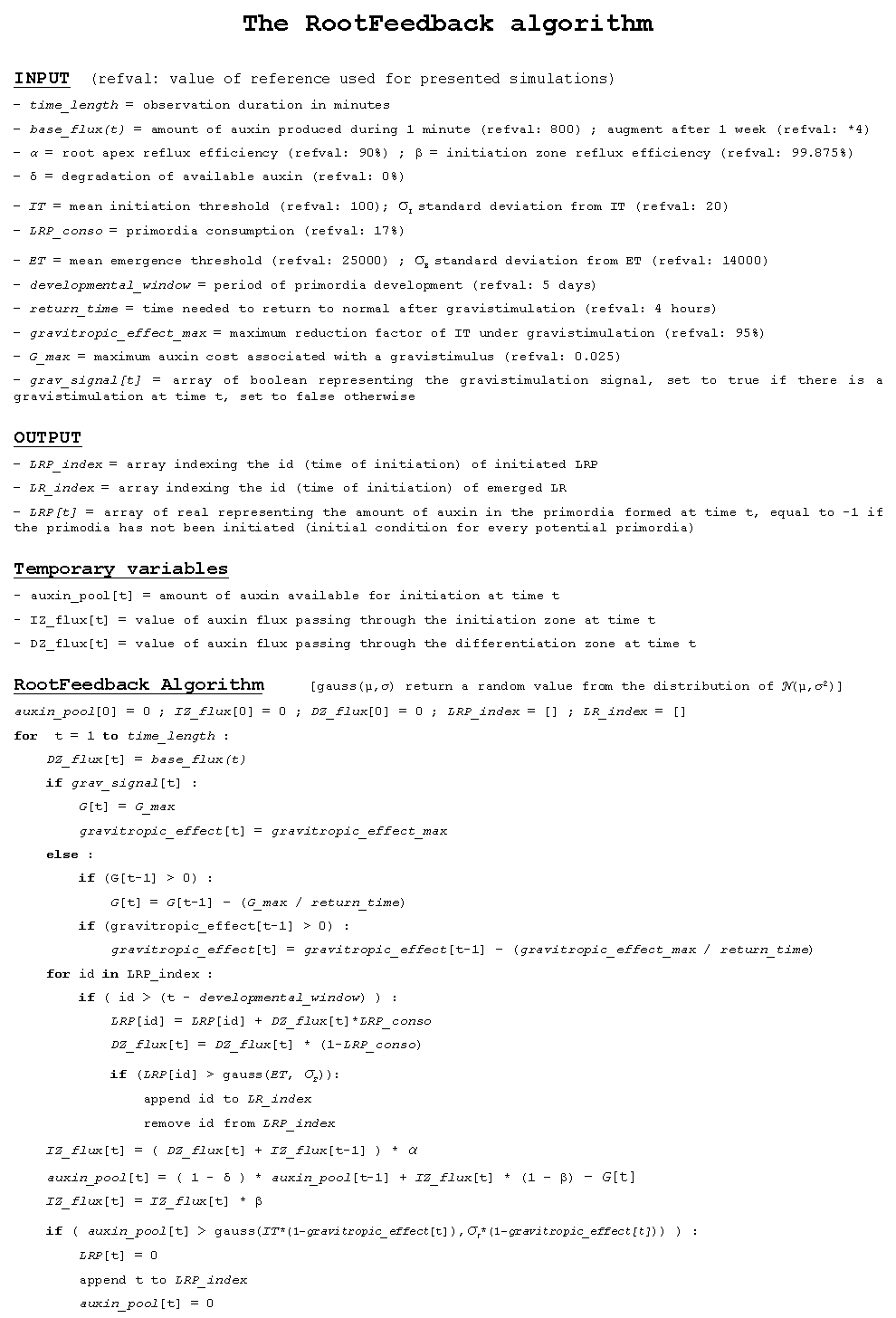

Supplement: Figure S2 — The RootFeedback algorithm corresponding to the mechanistic model The pseudo-code is expressing the mechanisms described in Fig. 8A in discrete time. (0.14 MB TIF) [file pone.0003673.s002.tif]

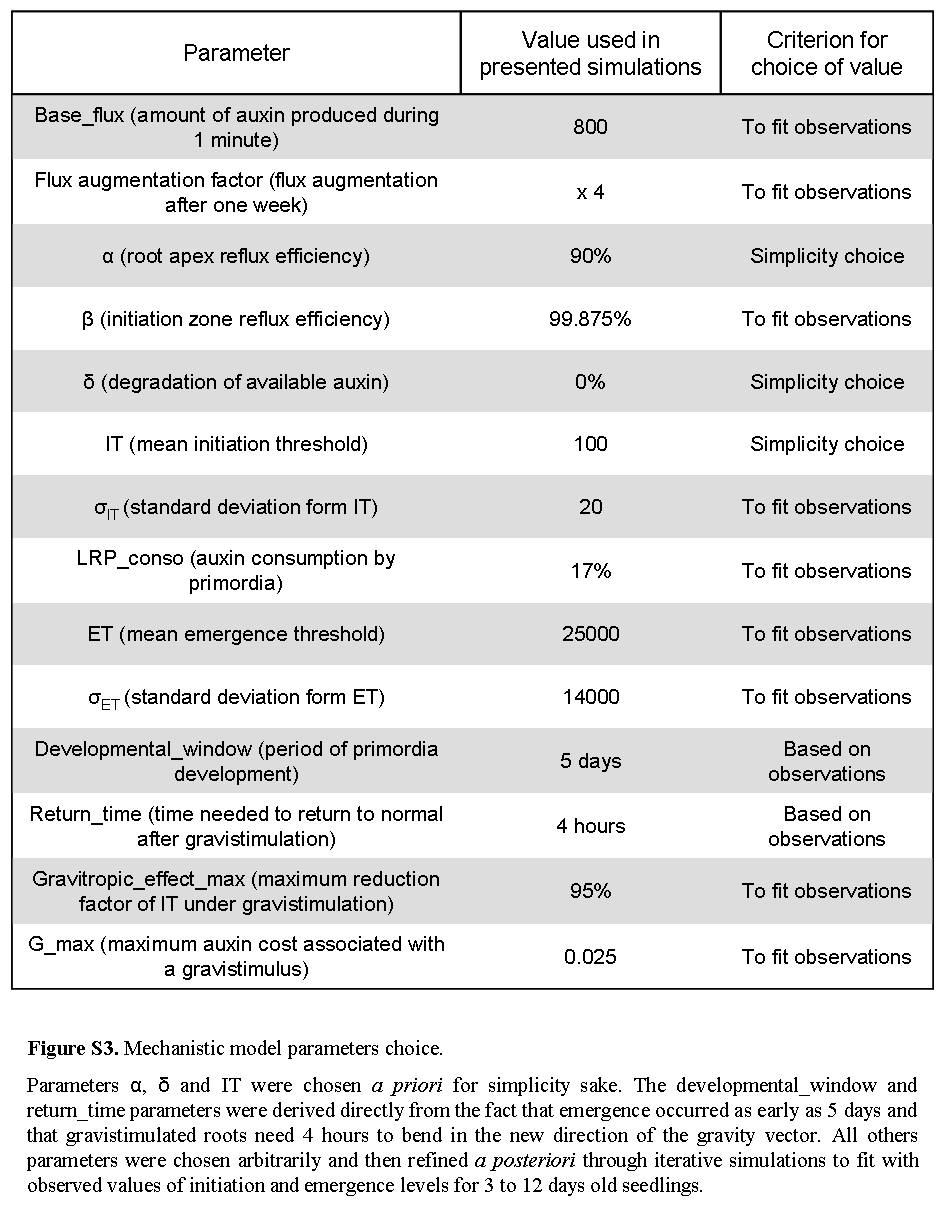

Supplement: Figure S3 — Mechanistic model parameters choice. Parameters α, δ and IT were chosen a priori for simplicity sake. The developmental_window and return_time parameters were derived directly from the fact that emergence occurred as early as 5 days and that gravistimulated roots need 4 hours to bend in the new direction of the gravity vector. All others parameters were chosen arbitrarily and then refined a posteriori through iterative simulations to fit with observed values of initiation and emergence levels for 3 to 12 days old seedlings. (0.17 MB TIF) [file pone.0003673.s003.tif]

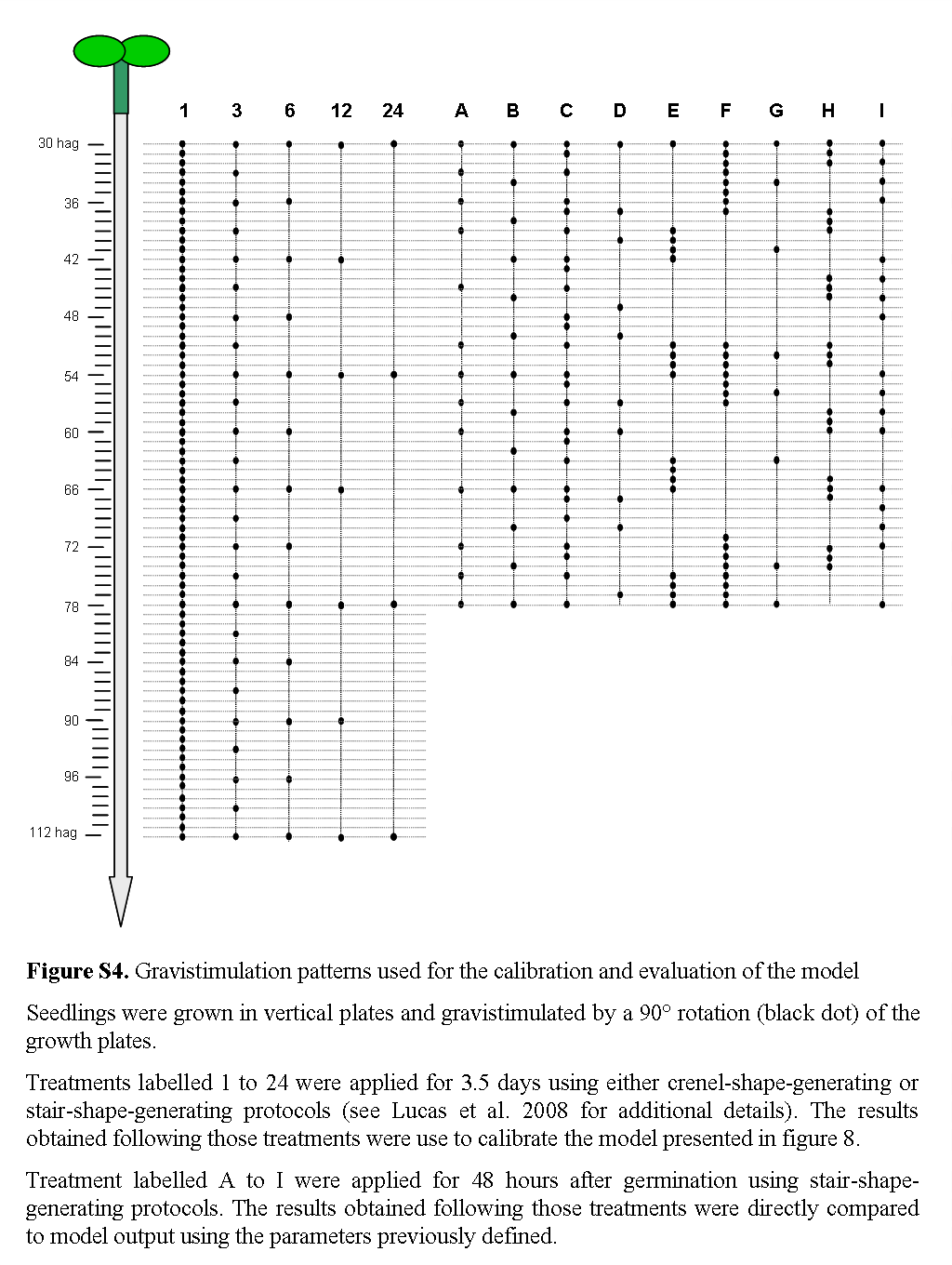

Supplement: Figure S4 — Gravistimulation patterns used for the calibration and evaluation of the model Seedlings were grown in vertical plates and gravistimulated by a 90° rotation (black dot) of the growth plates. Treatments labeled 1 to 24 were applied for 3.5 days using either crenel-shape-generating or stair-shape-generating protocols (see [15] for additional details). The results obtained following those treatments were use to calibrate the model presented in figure 8. Treatment labeled A to I were applied for 48 hours after germination using stair-shape-generating protocols. The results obtained following those treatments were directly compared to model output using the parameters previously defined. (0.27 MB TIF) [file pone.0003673.s004.tif]
